# PScL-2LSAESM: bioimage-based prediction of protein subcellular localization by integrating heterogeneous features with the two-level SAE-SM and mean ensemble method

**DOI:** 10.1093/bioinformatics/btac727

**Published:** 2022-11-22

**Authors:** Matee Ullah, Fazal Hadi, Jiangning Song, Dong-Jun Yu

**Affiliations:** School of Computer Science and Engineering, Nanjing University of Science and Technology, Nanjing 210094, China; School of Computer Science and Engineering, Nanjing University of Science and Technology, Nanjing 210094, China; Department of Biochemistry and Molecular Biology, Monash Biomedicine Discovery Institute, Monash University, Melbourne, VIC 3800, Australia; Monash Data Futures Institute, Monash University, Melbourne, VIC 3800, Australia; School of Computer Science and Engineering, Nanjing University of Science and Technology, Nanjing 210094, China

## Abstract

**Motivation:**

Over the past decades, a variety of *in silico* methods have been developed to predict protein subcellular localization within cells. However, a common and major challenge in the design and development of such methods is how to effectively utilize the heterogeneous feature sets extracted from bioimages. In this regards, limited efforts have been undertaken.

**Results:**

We propose a new two-level stacked autoencoder network (termed 2L-SAE-SM) to improve its performance by integrating the heterogeneous feature sets. In particular, in the first level of 2L-SAE-SM, each optimal heterogeneous feature set is fed to train our designed stacked autoencoder network (SAE-SM). All the trained SAE-SMs in the first level can output the decision sets based on their respective optimal heterogeneous feature sets, known as ‘intermediate decision’ sets. Such intermediate decision sets are then ensembled using the mean ensemble method to generate the ‘intermediate feature’ set for the second-level SAE-SM. Using the proposed framework, we further develop a novel predictor, referred to as PScL-2LSAESM, to characterize image-based protein subcellular localization. Extensive benchmarking experiments on the latest benchmark training and independent test datasets collected from the human protein atlas databank demonstrate the effectiveness of the proposed 2L-SAE-SM framework for the integration of heterogeneous feature sets. Moreover, performance comparison of the proposed PScL-2LSAESM with current state-of-the-art methods further illustrates that PScL-2LSAESM clearly outperforms the existing state-of-the-art methods for the task of protein subcellular localization.

**Availability and implementation:**

https://github.com/csbio-njust-edu/PScL-2LSAESM.

**Supplementary information:**

Supplementary data are available at *Bioinformatics* online.

## 1 Introduction

The knowledge regarding the precise subcellular location of a protein is crucial for the determination of its function and involved biological processes (Dallago *et al.*, 2017; Zhou *et al.*, 2017). Location proteomics is concerned with the large-scale study of protein localization inside a cell (Murphy, 2005; Newberg *et al.*, 2009). In location proteomics, various methods are used to analyze and predict protein subcellular localization such as wet-lab experiments or computational methods. Determining the protein subcellular localization via wet-lab experiments is often time-consuming and labor-intensive. Due to the importance of subcellular location of proteins and as a useful alternative to facilitate experimental characterization of protein subcellular localization, automatic computational methods are attracting a great deal of interest in recent years, representing the main focus in location proteomics.

Over the past decades, a great variety of computational methods have emerged for characterization of protein subcellular localization from diverse protein data sources, including amino acid sequences or bioimages. Based on the data sources, these methods can be generally categorized into either one-dimensional sequence-based or two-dimensional (2D) image-based methods which can be further grouped into single-label or multi-label methods (Cheng *et al.*, 2018; Hu *et al.*, 2022; Özsarı *et al.*, 2022; Xu *et al.*, 2018, 2020; Xue *et al.*, 2020; Yang *et al.*, 2014; Yu *et al.*, 2019; Zhang *et al.*, 2021). In the case of sequence-based methods, they can effectively determine the location of the protein (Chou and Shen, 2008; Guo *et al.*, 2016) and protein properties (Li *et al.*, 2021; Liu, 2019; Liu *et al.*, 2018; Luo *et al.*, 2019). However, as amino acid sequences do not change whenever the translocation takes place, this will make them unfit for the detection of subcellular translocation of proteins. In this regard, the image-based methods can serve as an alternative complementary to the sequence-based methods because they can unravel the crucial information regarding the spatial distribution of proteins across the normal and cancerous tissues, as well as their location changes in various tissues. Therefore, development of 2D image-based computational methods to facilitate the identification of protein subcellular localization has become an increasingly important problem in bioinformatics and computational biology (Peng *et al.*, 2012).

During the development of image-based computational methods for analyzing protein subcellular localization, considerable challenges often exist for statistical and machine learning models, especially related to feature extraction, feature selection (FS), feature integration and classification. For example, DNA distribution and Haralick texture features, which belong to subcellular location features (SLFs) (Boland and Murphy, 2001), are frequently utilized to represent and encode the global information from the bioimages. In addition, a variety of local features such as local binary pattern (LBP) (Ojala *et al.*, 2002), completed local binary pattern (CLBP) (Guo *et al.*, 2010), local ternary pattern (Tan and Triggs, 2007), local quinary pattern (Nanni *et al.*, 2010), rotation invariant co-occurrence among adjacent local binary patterns (RICLBP) (Nosaka *et al.*, 2013) and locally encoded transform feature histogram (LETRIST) (Song *et al.*, 2018) are utilized to extract the local micropatterns from images. Current studies have shown that extracting both global and local features can help improve the predictive capabilities of the developed methods (Xu *et al.*, 2013, 2016; Yang *et al.*, 2019). Similarly, at the FS stage, a number of studies have proposed different FS algorithms to effectively select the optimal features from the extracted features (Liu *et al.*, 2020; Newberg and Murphy, 2008; Shao *et al.*, 2018; Ullah *et al.*, 2021). Among such FS algorithms, the stepwise discriminant analysis (SDA) algorithm (Klecka, 1980) has been widely adopted and shown to be effective for FS.

Use of multiple heterogeneous features is a common step during the development of automated models as it remains a challenging task to represent the global and local features from images based on single traditional handcrafted feature sets. As such, the difficulty in feature integration arises when multiple features are used to represent the protein image. One useful way to utilize multiple feature sets is to simply concatenate all the feature sets in a simple serial fashion. Many protein subcellular localization prediction methods have utilized this simple serial integration strategy to integrate all the feature sets and subsequently develop a predictor (Tahir *et al.*, 2013; Ullah *et al.*, 2022; Xu *et al.*, 2016, 2018). More recently, several studies have investigated new techniques other than simple serial integration (Shao *et al.*, 2016; Wang *et al.*, 2022). However, less attention is being paid to integrating multiple feature sets and accordingly, there still remains significant challenges as to how these multiple feature sets can be efficiently integrated.

From the algorithmic perspective, a number of different classification models have been developed to predict protein subcellular localization. For example, support vector machine (Cortes and Vapnik, 1995), error correcting output coding (Dietterich and Bakiri, 1994), discriminant error correcting output coding (Pujol *et al.*, 2006), random forest (RF) (Breiman, 2001) and deep learning-based models (Hinton *et al.*, 2006; Hochreiter and Schmidhuber, 1997; Simonyan and Zisserman, 2015) have been utilized efficiently. Despite the extensive efforts being undertaken, currently available computational approaches continue to have insufficient and limited performance of protein subcellular localization. This is particularly the case in terms of the overall success rate and as a consequence, there remains an exigent need to develop novel and high-performance predictors.

Motivated by the issues mentioned above, in this study, we make the following contributions in order to improve the predictive performance of protein subcellular localization: first, to ensure that the dataset is up to date and no mistakenly labeled data are included, we collect the high-quality datasets from the latest version of human protein atlas (HPA) databank (Uhlén *et al.*, 2015) as the collection of the latest datasets is highly desirable for the development of accurate predictors; Second, we design and develop a new classifier called the stacked autoencoder-SoftMax (SAE-SM) network; Third, using the designed SAE-SM, we further develop a two-level SAE-SM (2L-SAE-SM) framework based on the integration of multiple feature sets, and fourth, based on 2L-SAE-SM, we implement a bioimage-based protein subcellular localization predictor termed PScL-2LSAESM. Benchmarking results on the stringent 10-fold cross-validation using the benchmark training dataset and the independent test using the independent test dataset illustrate the effectiveness of the proposed framework.

## 2 Materials and methods

### 2.1 Benchmark datasets

The benchmark image datasets used in this study were collected from the publicly available Tissue Atlas of the HPA database (version 21, http://proteinatlas.org) (Uhlen *et al.*, 2010; Uhlén *et al.*, 2015). The same criteria (i.e. reliability and validation scores) as Ullah *et al.* (2021) were considered during the collection of protein entries in our datasets. The immunohistochemistry (IHC)-based brightfield microscopic images of these proteins were collected in this study. All the images belong to normal human tissues; according to the annotations in HPA, each IHC image in the benchmark datasets was labeled as one of the seven major subcellular location classes including cytoplasm (Cytopl.), endoplasmic reticulum (ER), Golgi apparatus (Gol.), mitochondrion (Mito.), lysosome (Lyso.), nucleus (Nucl.) and vesicles (Vesi.).

The benchmark training dataset, referred to as PScL2708, encompasses 2708 IHC images belonging to 23 different proteins. The subcellular location classes including cytoplasm, endoplasmic reticulum, Golgi apparatus, mitochondrion, lysosome, nucleus and vesicles contain 4, 3, 3, 3, 2, 4 and 4 different proteins, respectively. Similarly, we also collected the independent test dataset called PScL227 in our study. PScL227 has 227 IHC images belonging to seven distinguished proteins. Each protein belongs to one subcellular location class. Table 1 summarizes the statistical distribution of the images across each subcellular location class for both the PScL2708 and PScL227 datasets.

**Table 1.** Statistical distribution of the images across each subcellular location class

Dataset	Subcellular location							Total
	Cytopl.	ER	Gol.	Lyso.	Mito.	Nucl.	Vesi	
PScL2708	483	310	345	224	374	472	500	2708
PScL227	34	36	27	36	30	34	30	227

### 2.2 Image separation and feature extraction

All IHC bioimages in the HPA database are the mixture of DNA and protein stains. As we were interested in the subcellular localizations of proteins only, therefore, we first used the linear spectral separation (LIN) method (see Supplementary Text S1 for details regarding the linear spectral separation) to separate each original IHC image into DNA and protein channels. Next, heterogeneous features extracted from multiple aspects might reveal hidden information from protein image samples, which is useful for predicting protein subcellular localization. Therefore, we extracted various global and local heterogeneous features considering that the global and local features are expected to extract complementary information from protein images (Yang *et al.*, 2014).

In our study, we extracted five types of heterogeneous feature sets from each IHC image. These included SLFs, LBP, CLBP, LETRIST and RICLBP with the dimensionalities of 840, 256, 906, 413 and 408, respectively. Previous studies have shown these features to be very effective in this field (Ullah *et al.*, 2021, 2022; Xu *et al.*, 2016). SLFs are the global features which includes 4-dimensional DNA distribution and 836-dimensional Haralick texture features (Boland and Murphy, 2001; Ullah *et al.*, 2022; Xu *et al.*, 2013). SLFs are very useful for extracting the global texture information from images (Boland and Murphy, 2001). The corresponding LBP features were extracted to characterize the local texture structure and detect micropatterns such as spots, edges and flat areas. In addition, CLBP and RICLBP features were also extracted to ensure the rotation invariance and information neglected by LBP. Similarly, LETRIST features were extracted because they could explicitly encode the joint information within the IHC image across the feature and scale spaces. For the sake of convenience, we termed LETRIST as LET in this study. A detailed description of SLFs, LBP, CLBP, LET and RICLBP is provided in Supplementary Text S2. In the current study, we accordingly named these five extracted heterogeneous feature sets as SLFs-Raw, LBP-Raw, CLBP-Raw, LET-Raw and RICLBP-raw, respectively.

### 2.3 Feature selection

In our study, all the five extracted heterogeneous features (i.e. SLFs-Raw, LBP-Raw, CLBP-Raw, LET-Raw and RICLBP-raw) have high dimensionalities and as such, there might exist irrelevant, redundant and noisy information which may either cause overfitting or underfitting. In order to avoid dimension explosion and remove feature redundancy, the original extracted features need to be reduced by some FS algorithms. A series of studies utilized various FS algorithms during the analysis of protein subcellular localization; however, among all these algorithms, the SDA has proven to be more effective. Therefore, in this study, we also employed the SDA algorithm on each feature set.

For a given training dataset $X={\left\{(x_{j};y_{j})\right\}}_{j=1}^{N}$, where $x_{j}$ is the *j*-th image sample, $y_{j}$ is its corresponding label and N is the total number of features, let $S_{t}={\left\{(x_{j,t};y_{j})\right\}}_{j=1}^{N}$ be the *t*-th (1≤t≤T) heterogeneous feature set extracted from X, where $x_{j,t}$ is the feature vector extracted from the *j*-th image sample for the *t-*th heterogeneous feature set. Suppose that for each feature vector *j* (i.e. $x_{j,t}$) in the *t*-th heterogeneous feature set $S_{t}$, $\left(x_{j,t}^{\mathrm{opt}};y_{i}\right)$ be its *j*-th data pair representing the *j*-th image sample of *X*, where $x_{j,t}^{\mathrm{opt}}\in R^{d_{t}^{\mathrm{opt}}}$ is the optimal feature vector for the *t*-th optimal heterogeneous feature set, $d_{t}^{\mathrm{opt}}$ is the dimension of the optimal feature vector. For Nnumber of features, a corresponding *t*-th optimal heterogeneous feature set, denoted as $S_{t}^{\mathrm{opt}}={\left\{(x_{j,t}^{\mathrm{opt}};y_{j})\right\}}_{j=1}^{N}$ can be generated.

For *T* heterogeneous feature sets $(i.e.\;S_{1}={\left\{(x_{j,1};y_{j})\right\}}_{j=1}^{N},$$S_{2}={\left\{(x_{j,2};y_{j})\right\}}_{j=1}^{N},\;\ldots ,\;S_{t}={\left\{(x_{j,t};y_{j})\right\}}_{j=1}^{N},\ldots ,\;S_{T}={\left\{(x_{j,T};y_{j})\right\}}_{j=1}^{N})$, a total of *T* optimal heterogeneous feature sets $(i.e.\;S_{1}^{\mathrm{opt}}={\left\{(x_{j,1}^{\mathrm{opt}};y_{j})\right\}}_{j=1}^{N},S_{2}^{\mathrm{opt}}={\left\{(x_{j,2}^{\mathrm{opt}};y_{j})\right\}}_{j=1}^{N},\;\ldots ,\;S_{t}^{\mathrm{opt}}={\left\{(x_{j,t}^{\mathrm{opt}};y_{j})\right\}}_{j=1}^{N},\;\ldots ,\;S_{T}^{\mathrm{opt}}={\left\{(x_{j,T}^{\mathrm{opt}};y_{j})\right\}}_{j=1}^{N})$ can be selected. For more theoretical and mathematical details of SDA algorithms, please refer to the Supplementary Text S3.

In our study, we represent the optimal heterogeneous feature set of SLFs-Raw as SLFs-optimal, LBP-Raw as LBP-Optimal, CLBP-Raw as CLBP-Optimal, LET-Raw as LET-Optimal and RICLBP-raw as RICLBP-Optimal, respectively.

### 2.4 Stacked autoencoder

A single autoencoder (AE) (Rumelhart *et al.*, 1986) consists of an input layer, a hidden layer and an output layer (for details about autoencoder, please refer to the Supplementary Text S4). In order to construct a stacked autoencoder (SAE), multiple AEs are stacked on top of each other. In other words, an SAE is a neural network consisting of multiple layers of AEs where the activation output features of the *k*-th hidden layer of AE are sent as an input to the (*k *+* *1)-th hidden layer of AE. In cases where an SAE is used as a classifier, a classification layer must be added as the top layer to compute and output the probabilities of the classes. The purpose of stacking multiple AEs is to boost the performance of the model.

**Fig. 1.**
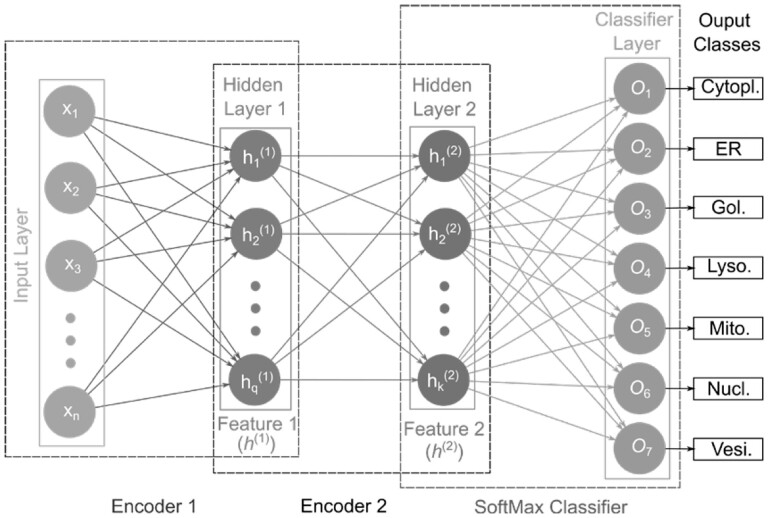
Illustration of the SAE-SM-model architecture

In the current study, we used two encoders and the SoftMax (SM) activation function as a classification layer to construct our stacked autoencoder network, referred to as SAE-SM as shown in Figure 1. Given the input x (Input Layer), the first encoder (Encoder 1) produces first hidden layer activation output features $h^{\left(1\right)}$ (Features 1). The activation output features $h^{\left(1\right)}$will be then fed to the second encoder (i.e. Encoder 2) generating the second hidden layer activation output features $h^{\left(2\right)}$ (i.e. Features 2). The activation output features $h^{\left(2\right)}$ are finally fed to the SM classifier layer (SoftMax Classifier) to output the corresponding class probabilities. Each hidden layer size and hyperparameters are provided in Supplementary Text S5. In the hidden layers, we used the sigmoid activation function and also imposed the sparsity constraint on hidden units. Two training phases are involved during the training of our SAE-SM: (i) layer-by-layer pre-training which uses the unsupervised learning method and (ii) fine-tuning which uses the supervised back propagation (BP) method. For example, once the first AE is pre-trained based on the input x, the output ($h^{\left(1\right)}$) of the first AE can then be input to the next AE. This procedure continues until the pre-training is accomplished (i.e. layer-by-layer pre-training). Finally, the pre-trained SAE-SM is fine-tuned using the BP algorithm (i.e. fine-tuning).

### 2.5 Proposed 2L-SAE-SM

**Fig. 2.**
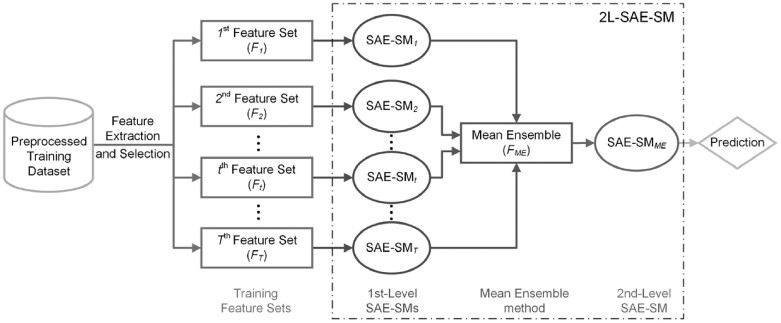
The architecture of the proposed 2L-SAE-SM model for integrating heterogeneous feature sets

In order to effectively integrate multiple feature sets, we propose the 2L-SAE-SM framework. Figure 2 illustrates its architecture. As can be seen, 2L-SAE-SM is a two-level model where in the first level, T number of SAE-SMs, denoted as SAE-SM_*1*_, SAE-SM_*2*_, …, SAE-SM_*T*__-1_, SAE-SM_*T*_, are trained on the T optimal heterogeneous feature sets selected from the T raw heterogeneous feature sets via the SDA algorithm detailed in the Section 2.3 to further learn the hidden information from the corresponding optimal heterogeneous feature set. The Mean Ensemble (ME) method is applied in the middle of two levels to ensemble the outputs of the trained first-level SAE-SMs, whose output would be fed into the second-level SAE-SM_*ME*_ for making the prediction.

Next, we describe the ME in detail below:

Let ${\left\{p_{t}\right\}}_{t=1}^{T}$ be the T ‘intermediate decision’ sets, where $p_{t}$ denotes the *t*-th ‘intermediate decision’ set. Then the ME can be represented as:

(1) $$F_{ME}=\frac{1}{T}\sum_{t=1}^{T}p_{t}$$

where $F_{ME}$ denotes the ‘intermediate feature’ set.

A major challenge is how to train a 2L-SAE-SM on a given training dataset. In order to efficiently handle this issue, Supplementary Text S6 provides a detailed description of the training procedures of 2L-SAE-SM.

**Fig. 3.**
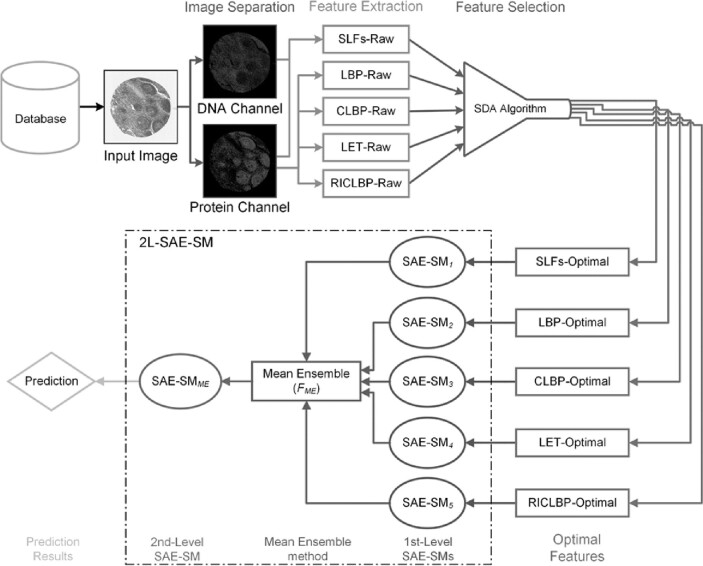
Illustration of the architecture of the proposed PScL-2LSAESM model

Based on the 2L-SAE-SM framework, we developed a novel predictor PScL-2LSAESM to characterize image-based protein subcellular localization. An overview of the working flow of the proposed PScL-2LSAESM is illustrated in Figure 3. Each major stage of the PScL-2LSAESM is described in Supplementary Text S7 and the system configuration settings are discussed in Supplementary Text S8.

### 2.6 Evaluation indices

In this study, five commonly used performance indices specially designed for evaluating the performance of multiclass learning are employed. These included the overall accuracy (OA), Macroaverage Precision (Prec_M_), Macroaverage Recall (Rec_M_), Macroaverage F1-Score (F1-Score_M_) and Matthews’ Correlation Coefficient (MCC). In addition to these indices, the mean of the area under the receiver-operating characteristic (ROC) curves (AUC) denoted as meanAUC, the mean of area under precision-recall (PR) curves (AUPR) denoted as meanAUPR and the standard deviation of AUC and AUPR denoted as stdAUC and stdAUPR, respectively, are also used as the other four evaluation indices. All the performance evaluation indices are described in detail in the Supplementary Text S9.

Stringent *k*-fold cross-validation and independent validation tests are conducted to evaluate the performance of the proposed model. When performing *k*-fold cross-validation, the value of *k* was set to 10. It is noteworthy that when evaluating the performance of the model via stringent *k*-fold cross-validation, the features were selected independently in each fold of the train dataset to avoid biased evaluation of the model performance.

## 3 Results

### 3.1 Performance comparison of the extracted heterogeneous feature sets

In this section, we examined the discriminative capabilities of SLFs-Raw, LBP-Raw, CLBP-Raw, RICLBP-Raw and LET-Raw heterogeneous feature sets. The performance of each heterogeneous feature set was evaluated by performing 10-fold cross-validation on the benchmark training dataset PScL2708 with our designed SAE-SM classifier. The performance comparison of all the five heterogeneous feature sets in terms of OA, Rec_M_, Prec_M_, F1-Score_M_ and MCC is provided in Table 2.

**Table 2.** Performance comparison of the raw feature sets on 10-fold cross-validation using the benchmark training dataset PScL2708

Feature set	OA (%)	Rec_M_ (%)	Prec_M_ (%)	F1-Score_M_	MCC
SLFs-Raw	67.54	65.90	66.66	0.6622	0.6168
LBP-Raw	80.90	79.54	80.07	0.7977	0.7748
CLBP-Raw	81.83	80.66	81.06	0.8084	0.7857
RICLBP-Raw	78.84	77.69	77.93	0.7775	0.7506
LET-Raw	77.80	76.42	76.88	0.7662	0.7382

Several observations can be derived from Table 2: first, among all the heterogeneous feature sets, the CLBP-Raw heterogeneous feature set served as the best performer in terms of all the evaluation metrics, suggesting the superiority of CLBP-Raw over the other four heterogonous feature sets. For example, CLBP-Raw achieved the F1-Score_M_ = 0.8084 and MCC = 0.7857, which were 14.62% and 16.89%, 1.07% and 1.09%, 3.09% and 3.51%, and 4.22% and 4.75% higher than SLFs-Raw, LBP-Raw, RICLBP-Raw and LET-Raw, respectively. Upon closer inspection of the other four heterogeneous feature sets, we found that the LBP-Raw and RICLBP-Raw achieved the second and third best performance, respectively; Second, the performance of SLFs-Raw was not satisfactory. A possible reason is that the extracted raw heterogeneous feature sets might have redundant and noisy information which can result in the decreased predictive capabilities of the model; Third, keeping in mind that all the extracted raw heterogeneous feature sets may have redundant and noisy information, the performance results in Table 2 suggest that all the five heterogeneous feature sets examined in this study can be effectively used to predict protein subcellular localization.

### 3.2 Performance comparison of optimal heterogeneous feature sets

As described in the Section 2.3, we fed each of the five extracted heterogeneous feature sets (i.e. SLFs-Raw, LBP-Raw, CLBP-Raw, RICLBP-Raw and LET-Raw) into the SDA FS algorithm and obtained its corresponding optimal heterogeneous feature set. Next, to investigate the discriminative capability of each optimal heterogeneous feature set, we performed 10-fold cross-validation on PScL2708 with SAE-SM as the classifier. Table 3 provides the performance results of all the five selected optimal heterogeneous feature sets (i.e. SLFs-Optimal, LBP-Optimal, CLBP-Optimal, RICLBP-Optimal and LET-Optimal) in terms of OA, Rec_M_, Prec_M_, F1-Score_M_ and MCC.

**Table 3.** Performance comparison of the optimal feature sets on 10-fold cross-validation using the benchmark training dataset PScL2708

Feature set	OA (%)	Rec_M_ (%)	Prec_M_ (%)	F1-Score_M_	MCC
SLFs-Optimal	77.62	76.72	76.51	0.7660	0.7363
LBP-Optimal	83.19	82.23	82.11	0.8215	0.8020
CLBP-Optimal	84.08	82.83	83.02	0.8292	0.8123
RICLBP-Optimal	79.91	79.14	79.89	0.7940	0.7631
LET-Optimal	80.35	79.26	79.39	0.7932	0.7683

**Fig. 4.**
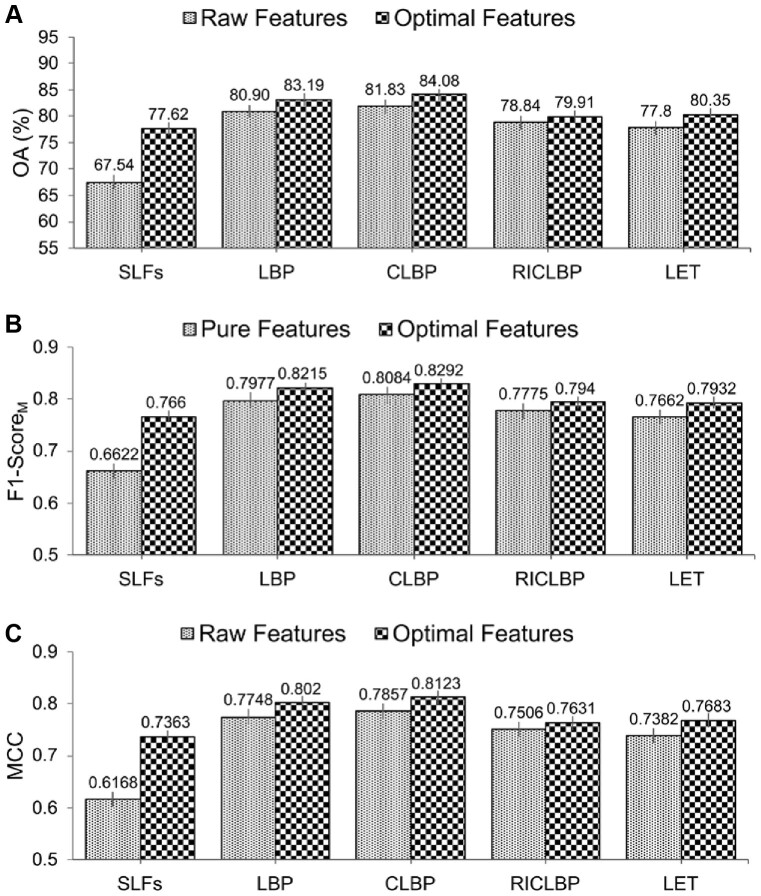
Performance comparison between the raw and optimal heterogeneous feature sets. Panels (**A–C**) display the performance comparisons in terms of OA, F1-ScoreM and MCC, respectively

From Table 3, it can be observed that use of the SDA FS algorithm indeed helped improve the predictive performance of protein subcellular localization. Particularly, for each of the optimal heterogeneous feature sets, all the evaluation metrics (i.e. OA, Rec_M_, Prec_M_, F1-Score_M_ and MCC) were improved compared with the raw heterogeneous feature sets. Moreover, among all the extracted raw and optimal heterogeneous feature sets, CLBP-Optimal consistently achieved the maximal performance of OA, Rec_M_, Prec_M_, F1-Score_M_ and MCC, which were 84.08%, 82.83%, 83.02%, 0.8292 and 0.8123, respectively. For a fair comparison, Figure 4A–C show the bar-graph representations of the OA, F1-Score_M_ and MCC values of the raw and optimal heterogeneous feature sets. We can see that SAE-SM trained using the optimal heterogeneous feature sets consistently achieved a better performance than its counterpart trained using the raw heterogeneous feature sets. More specifically, from Figure 4A, it can be seen that the predictive performance of the SAE-SM in term of OA on SLFs-Optimal, LBP-Optimal, CLBP-Optimal, RICLBP-Optimal and LET-Optimal was improved by 10.07%, 2.29%, 2.25%, 1.07% and 2.55%, respectively, in comparison to that of the SAE-SM achieved on SLFs-Raw, LBP-Raw, CLBP-Raw, RICLBP-Raw and LET-Raw. Similarly, as shown in Figure 4B and C, the F1-Score_M_ and MCC values were also improved based on the optimal heterogeneous feature sets.

In summary, the results in Table 3 and Figure 4A–C clearly demonstrate that utilizing the optimal heterogeneous feature sets consistently improved the predictive performance of our proposed SAE-SM method. Therefore, in the following sections, instead of utilizing the raw heterogeneous feature sets, we used SLFs-Optimal, LBP-Optimal, CLBP-Optimal, RICLBP-Optimal and LET-Optimal as our five optimal heterogeneous feature sets to construct our model.

### 3.3 Integrating feature sets via 2L-SAE-SM to improve the prediction performance

Supplementary Text S10 provides the performance comparison of different classifiers based on serial integration of the optimal heterogeneous feature sets. In this section, we seek to examine the effectiveness of the proposed 2L-SAE-SM method and investigate whether integrating all the optimal heterogeneous feature sets with 2L-SAE-SM can be even more effective than the direct serial integration for predicting protein subcellular localization. To address this, we performed experiments on the PScL2708 using 10-fold cross-validation and the performance comparisons between the best optimal heterogeneous feature set, the serially integrated feature set and the feature set integrated by 2L-SAE-SM are provided in Table 4.

**Table 4.** Performance comparison between the best optimal features, features integrated serially and by 2L-SAE-SM on 10-fold cross-validation using the benchmark training dataset PScL2708

Method	OA (%)	Rec_M_ (%)	Prec_M_ (%)	F1-Score_M_	MCC
CLBP-Optimal+SAE-SM	84.08	82.83	83.02	0.8292	0.8123
Serial+SAE-SM	87.18	86.52	86.56	0.8653	0.8489
Features integrated by 2L-SAE-SM	90.25	89.31	89.84	0.8953	0.8851

From Table 4, we can readily observe that the optimal heterogeneous feature sets integrated by 2L-SAE-SM drastically improved the performance. Compared with the feature set obtained by direct serial integration, the feature set integrated by 2L-SAE-SM achieved 3.07%, 3% and 3.62% improvements OA, F1-Score_M_ and MCC, respectively. Similarly, by comparing with the CLBP-Optimal feature set, the feature set integrated by 2L-SAE-SM showed improvements of 6.17%, 6.61% and 7.28% in OA, F1-Score_M_ and MCC, respectively. Additionally, the feature set integrated by 2L-SAE-SM also consistently performed well in terms of Rec_M_ and Prec_M_.

**Fig. 5.**
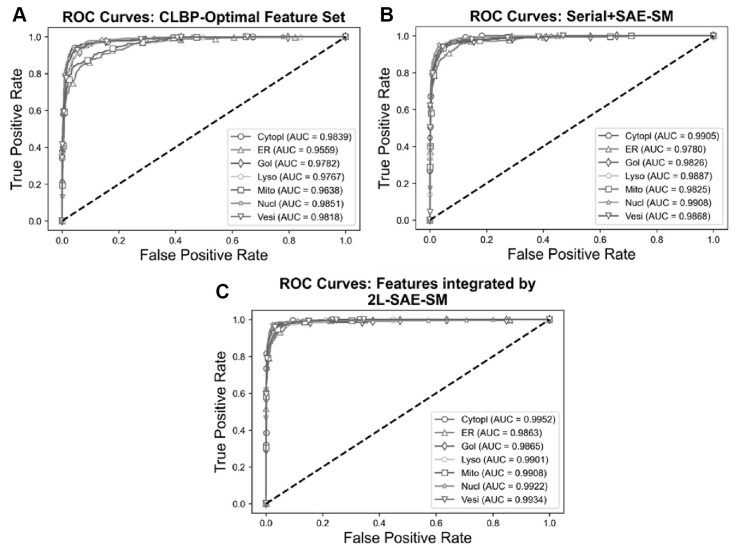
ROC curves of the optimal heterogeneous features and features integrated serially and by 2L-SAE-SM. Panel (**A**) shows the ROC curves for the CLBP-Optimal feature set, panel (**B**) shows the ROC curves for the serially integrated feature set and panel (**C**) shows the ROC curves

**Fig. 6.**
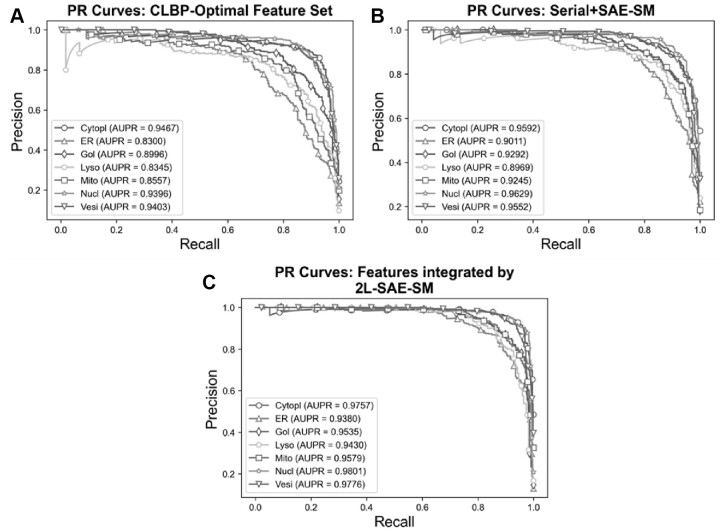
PR curves for optimal heterogeneous feature, features integrated serially and by 2L-SAE-SM. Panel (**A**) shows the PR curves for the CLBP-Optimal feature set, panel (**B**) shows the PR curves for the serially integrated feature set and panel (**C**) shows the PR curves for the feature set integrated by 2L-SAE-SM

In order to verify the efficacy of the proposed 2L-SAE-SM, we compared the performance of these three feature sets in terms of ROC and PR curves in Figures 5A–C and 6A–C. In particular, Figures 5A and 6A, 5B and 6B and 5C and 6C show the ROC and PR curves for the CLBP-Optimal feature set, the serially integrated feature set and the feature set integrated by 2L-SAE-SM, respectively. Compared with the CLBP-Optimal and serially integrated feature sets, the feature set integrated by 2L-SAE-SM consistently achieved better performance by improving the ROC curve and AUC value for each individual class as shown in Figure 5A–C. In terms of meanAUC, feature set integrated by 2L-SAE-SM achieved the meanAUC of 0.9906 and stdAUC of 0.0033 which was improved by 1.56% and 0.49% in comparison to the meanAUCs of 0.9750 and 0.9857 achieved by CLBP-Optimal and serially integrated feature sets, respectively. In addition, the feature set integrated by 2L-SAE-SM achieved the stdAUC of 0.0033 which was decreased by 0.77% and 0.15% in compassion to the stdAUCs of 0.0110 and 0.0048 achieved by CLBP-Optimal and serially integrated feature sets, respectively. Similarly, from Figure 6A–C, the feature set integrated by 2L-SAE-SM achieved improvements in terms PR curve of AUPR value for each individual class. Considering the meanAUPR and stdAUPR, the feature set integrated by 2L-SAE-SM achieved the meanAUPR and stdAUPR of 0.9608 and 0.0171, respectively, which is clearly better than the meanAUPR and stdAUPR of 0.8923 and 0.0518 achieved by CLBP-Optimal feature set and the meanAUC and stdAUPR of 0.9326 and 0.0273 achieved by serially integrated feature set. Altogether, the results and performance comparisons in terms of all evaluation indices in Table 4, Figures 5A–C and 6A–C prove that integrating all the optimal feature sets by 2L-SAE-SM can indeed improve the prediction accuracy for protein subcellular localization.

Based upon the proposed 2L-SAE-SM, we developed a new computational method termed PScL-2LSAESM for the prediction of protein subcellular localization.

### 3.4 Performance comparison with the other existing methods

In this section, to further illustrate the predictive power of PScL-2LSAESM, we performed experiments and compared its performance with that of the other existing protein subcellular localization predictors including PScL-DDCFPred (Ullah *et al.*, 2022), PScL-HDeep (Ullah *et al.*, 2021), SAE-RF (Liu *et al.*, 2020), SC-PSorter (Shao *et al.*, 2016) as well as the method proposed by Yang *et al.* (2014).

#### 3.4.1 Performance comparison on 10-fold cross-validation test

**Fig. 7.**
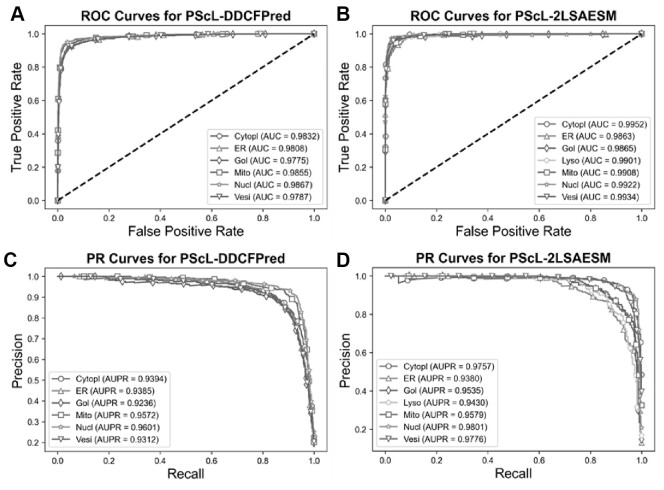
Performance comparisons between PScL-DDCFPred and the proposed PScL-2LSAESM method. Panels (**A**) and (**B**) show the ROC Curves, while (**C**) and (**D**) show the PR curves of PScL-DDCFPred and PScL-2LSAESM, respectively

In this section, we further compared the proposed PScL-2LSAESM with the other existing predictors by conducting 10-fold cross-validation test on the PScL2708 dataset. To show the predictive capability, we first compared PScL-2LSAESM with the most recently published PScL-DDCFPred predictor. Table 5 and Figure 7A–D show the experimental results of the two predictors.

**Table 5.** Performance comparison between PScL-2LSAESM and PScL-DDCFPred on 10-fold cross-validation using the benchmark training dataset PScL2708

Method	OA (%)	Rec_M_ (%)	Prec_M_ (%)	F1-Score_M_	MCC
PScL-DDCFPred^a^	88.40	88.38	88.54	0.8839	0.8609
PScL-2LSAESM	90.25	89.31	89.84	0.8953	0.8851

a Results were calculated using the source code and data of PScL-DDCFPred.

From Table 5 and Figure 7A–D, we conclude that the proposed PScL-2LSAESM outperformed the recently published PScL-DDCFPred. In particular, PScL-2LSAESM achieved the OA, F1-Score_M_ and MCC of 90.25%, 0.8953 and 0.8851, respectively, which were improved by 1.85%, 1.14% and 2.24% compared with the OA, F1-Score_M_ and MCC of the PScL-DDCFPred predictor, respectively. In addition, the Rec_M_ and Prec_M_ of the PScL-2LSAESM were consistently improved as well.

Further, upon close inspection of Figure 7A and C which show the ROC and PR curves of PScL-DDCFPred and Figure 7B and D which show the ROC and PR curves of PScL-2LSAESM, respectively, we can see that the performance of the proposed PScL-2LSAESM was also clearly improved in terms of both ROC and PR curves. As shown in Figure 7B, the AUC values achieved by PScL-2LSAESM were consistently improved across all the subcellular localization classes compared with those of PScL-DDCFPred shown in Figure 7A. Similarly, upon a closer look at the results in Figure 7D, we can see that the proposed PScL-2LSAESM also achieved better AUPR curve values for the majority of the subcellular localization classes in comparison to the results in Figure 7C for PScL-DDCFPred. In addition, the meanAUC and meanAUPR values of the recently published PScL-DDCFPred were 0.9821 and 0.9417, respectively, while the meanAUC and meanAUPR values of the proposed PScL-2LSAESM were 0.9906 and 0.9608, respectively, the latter of which was clearly improved. Moroever, PScL-DDCFPred attained the stdAUC and stdAUPR values of 0.0037 and 0.0144, while the proposed PScL-2LSAESM method attained the stdAUC and stdAUPR of 0.0033 and 0.0171. Although the stdAUPR value of the proposed PScL-2LSAESM was not improved compared with PScL-DDCFPred, it achieved a competitive performance. In summary, the performance improvements in terms of all the other evaluation indices including OA, Rec_M_, Prec_M_, F1-Socre_M_, MCC, ROC curves and its AUC, meanAUC and stdAUC values, PR curves and its AUPR, meanAUPR values suggest the superiority of the proposed PScL-2LSAESM method over the previously developed PScL-DDCFPred method.

Next, we benchmarked the predictive capability of the proposed PScL-2LSAESM against that of PScL-HDeep, SAE-RF, SC-PSorter and Yang *et al.’s* method in Table 6 in terms of OA, meanAUC and stdAUC. We can see that PScL-2LSAESM consistently achieved a better performance with an improvement of about 4.3–12.63% and 0.88–2.45% in OA and meanAUC, respectively, than the OA and meanAUC values of the other existing predictors. Similarly, the stdAUC of the PScL-2LSAESM was also the lowest.

**Table 6.** Performance comparison of PScL-2LSAESM and the other existing methods on 10-fold cross-validation using the benchmark training dataset PScL2708

Method	OA (%)	meanAUC	stdAUC
Yang *et al.*^a^	77.62	0.9661	0.0229
SC-PSorter^a^	80.45	0.9702	0.0193
SAE-RF^a^	81.76	0.9715	0.0185
PScL-HDeep^b^	85.95	0.9818	0.0046
PScL-2LSAESM	90.25	0.9906	0.0033

a Data excerpted from Liu *et al.* (2020).

b Data excerpted from Ullah *et al.* (2021).

In conclusion, the benchmarking results on the PScL2708 dataset confirmed that the proposed PScL-2LSAESM method achieved the best performance for the prediction of single-label multiclass protein subcellular localization.

#### 3.4.2 Performance comparison on the independent test

In this section, we performed independent test to further assess the generalization capability of the proposed PScL-2LSAESM. For this purpose, we first trained PScL-2LSAESM on the PScL2708 dataset and then tested the performance of the trained PScL-2LSAESM model on the independent PScL227 dataset. Next, we compared the performance of PScL-2LSAESM with that of the recently published PScL-DDCFPred in terms of OA, Rec_M_, Prec_M_, F1-Score_M_ and MCC. The performance results are provided in Table 7.

**Table 7.** Performance comparison between PScL-2LSAESM and PScL-DDCFPred on the independent test dataset PScL227

Method	OA (%)	Rec_M_ (%)	Prec_M_ (%)	F1-Score_M_	MCC
PScL-DDCFPred^a^	72.28	72.30	74.41	0.7270	0.6694
PScL-2LSAESM	74.88	74.94	75.48	0.7471	0.7085

a Data excerpted from Ullah *et al.* (2022).

From Table 7, we can see that the proposed PScL-2LSAESM method achieved an improved performance than PScL-DDCFPred in terms of all the evaluation indices. For example, PScL-2LSAESM achieved the OA, F1-Score_M_ and MCC of 74.88%, 0.7471 and 0.7085, respectively, which were 2.6%, 2.01% and 3.91% higher than the OA, F1-Score_M_ and MCC values of PScL-DDCFPred, respectively. These results highlight that PScL-2LSAESM outperformed PScL-DDCFPred and provided a better generalization capability.

**Fig. 8.**
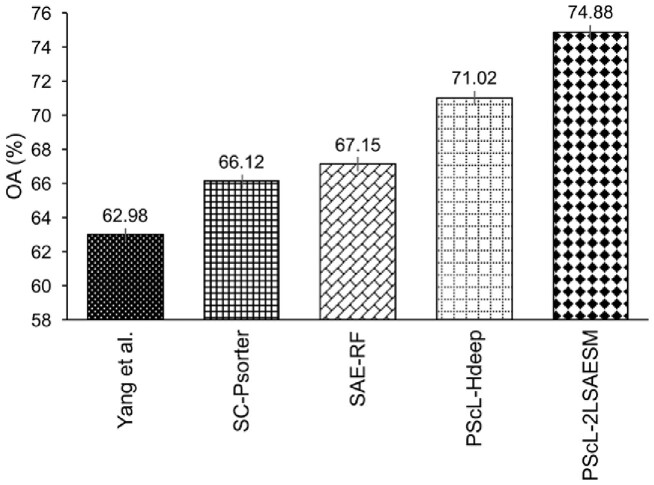
Performance comparison between PScL-2LSAESM and the existing predictors on the independent test dataset

To further illustrate the generalization capability of PScL-2LSAESM, we provide the performance comparison of the proposed PScL-2LSAESM, PScL-HDeep, SAE-RF, SC-PSorter and Yang *et al.’s* method in terms of OA in Figure 8. Note that parts of the results in Figure 8 were excerpted from the previous works of Liu *et al.* (2020) and Ullah *et al.* (2021). We can see that PScL-2LSAESM attained the OA of 74.88% which was 11.9%, 8.76%, 7.73% and 3.86% higher than that of Yang *et al.’s* method, SC-PSorter, SAE-RF and PScL-HDeep, respectively. From Figure 8, we conclude that PScL-2LSAESM has the best generalization capability compared with the other compared methods.

## 4 Discussion

In this study, we have proposed a new computational method termed PScL-2LSAESM to effectively improve the bioimage-based prediction of human protein subcellular localization in human tissues. Specifically, a 2L-SAE-SM system was developed to integrate multiple heterogeneous feature sets into PScL-2LSAESM. For this purpose, we first designed a stacked based autoencoder with the SoftMax as the classifier layer, referred to as SAE-SM. Using SAE-SM, we implemented a two-level SAE-SM (2L-SAE-SM) where in the first level, each of the optimal heterogeneous feature sets (i.e. SLFs-Optimal, LBP-Optimal, CLBP-Optimal, LET-Optimal and RICLBP-Optimal) was fed into a single SAE-SM and then output the decision level set termed the ‘intermediate decision’ set. All the produced intermediate decision sets were then integrated using the ME method in the 2L-SAE-SM as the ‘intermediate feature’ set and then sent to the second-level SAE-SM. Both stringent 10-fold cross-validation test on the newly collected benchmark training dataset PScL2708 and independent test on the newly collected independent test dataset PScL227 have demonstrated the effectiveness of the proposed 2L-SAE-SM method for heterogeneous feature set integration. Extensive benchmarking experiments have also shown that the proposed PScL-2LSAESM predictor clearly outperformed the other existing single-label multiclass protein subcellular localization prediction methods. We expect that the proposed predictor can be explored as a useful method to facilitate the characterization of single-label multiclass protein subcellular localizations. In the future work, we plan to develop improved strategies to improve the performance of the proposed framework through the integration of multiple data sources such as protein amino acid sequences, protein images and protein-protein interaction networks.

## Supplementary Material

btac727_Supplementary_Data

## Data Availability

All the data and source codes used in this study are freely available at https://github.com/csbio-njust-edu/PScL-2LSAESM.
