# Supplementary material for: PScL-2LSAESM: bioimage-based prediction of protein subcellular localization by integrating heterogeneous features with the two-level SAE-SM and mean ensemble method
Source: Bioinformatics. 2022 Nov 22;39(1):btac727. doi: 10.1093/bioinformatics/btac727 (PMC9947927; doi:10.1093/bioinformatics/btac727)
Supplement: btac727_Supplementary_Data [file btac727_supplementary_data.docx]

**PScL-2LSAESM: bioimage-based prediction of protein subcellular localization by integrating heterogeneous features with the two-level SAE-SM and mean ensemble method**

Matee Ullah^1^, Fazal Hadi^1^, Jiangning Song^2,3^,*, Dong-Jun Yu^1^,*

^1^School of Computer Science and Engineering, Nanjing University of Science and Technology, 200 Xiaolingwei, Nanjing, 210094, China, ^2^Monash Biomedicine Discovery Institute and Department of Biochemistry and Molecular Biology, Monash University, Melbourne, VIC 3800, Australia, ^3^Monash Data Futures Institute, Monash University, Melbourne, VIC 3800, Australia

*To whom correspondence should be addressed.

# **Text S1. Linear spectral separation**

The LIN spectral separation technique works when 1) there is a color basis available for each image and 2) the images are linearly separable. The LIN can formally be denoted by

$$I_{orignal}=V_{separated}\times H$$

where $I_{orignal}$is the original IHC bioimage; $V_{separated}$ is the vector after LIN spectral separation which contains two vectors of DNA and Protein channels; $H$ is the color basis.

In order to find the color basis $H$ in our study, a step-by-step process, discussed below, is followed. We first changed the IHC image white background to black. The image is then represented in the original form of $(r\times c)\times l$ matrix, where $r$, $c$ and $l$ are the rows, columns and color channels, respectively. $l$ is 3 representing number of colors in RGB space. In the Next step, we first transformed the IHC image into HSV color space and then calculated histogram in hue space. A hue threshold is defined and histogram bins greater than are equal to hue threshold are used to denote the hues of brown and less than hue threshold are used to define the hues of purple. We then calculated the corresponding brightness and saturation values for each hue. After that, mapping the HSV coordinates into RGB color space gave us two vectors $t_{1}$ and $t_{2}$ of stain color coefficients. These color coefficients show the measure of blue, green and red in each stain. Finally, we got color basis matrix for each IHC image by combining $t_{1}$ and $t_{2}$ vectors. The hue threshold was set to 0.3 in this study.

As we have a total of 23 antibodies. Therefore, we obtained the uniform color basis matrix $H$ by using the following formula:

$$H=\frac{1}{N}\sum_{j=1}^{23} N_{j}H_{j}^{antibody}$$

where $N_{j}$ is the total number of IHC images in the $j^{th}$ antibody; $H_{j}^{antibody}$ is the average color base matrix for the $j^{th}$ antibody; $N$ is the total number of IHC images;

After the color basis matrix $H$ is obtained, we then separated the channels using:

$$V_{seperated}=I_{original}\times\text{pinv}\left(H\right)$$

where $\text{pinv}\left(H\right)$ is the Moore-Penrose pseudo inverse (pinv) of color basis matrix $H$. The intensity range of $V_{seperated}$ was scaled into a range of 0 to 256 gray levels.

# **Text S2. Image-based features representation**

## **Subcellular location features**

Subcellular location features (SLFs) are the global features which includes DNA distribution and Haralick texture features in this study. The details of each are provided below:

### **DNA distribution features**

The significant dissimilarity between eukaryote and prokaryote cells is the membranous enclosed organelle nucleus. As human belongs to the former, each bioimage will have DNA staining. The DNA spatial distribution has been evidenced in previous studies to be valuable for improving the classification accuracy (Newberg and Murphy, 2008). Thus, here we also extracted the following four types of DNA-protein overlapping features:

- Ratio of the overall sum of pixel values in protein segment to DNA segment;
- Ratio of those number of pixels in the protein segment that co-localize with the DNA segment to the number of pixels in the protein segment;
- Ratio of the overall sum of pixel values in the protein segment that co-localize with the DNA region to the sum of pixels in the protein segment;
- Average distance between the protein segments that overlap with the DNA region and the nearest nuclear pixel.

### **Haralick texture features**

After linear color separation, we extracted the haralick texture features. These features were obtained through gray level co-occurrence matrix (GLCM). The GLCM was obtained via $N$ number of gray levels in the image constructed on a fixed angle $\theta$ and the measurement of the pixel distance $d$. In 2-dimensional square pixel image, the haralick texture features can be extracted from the four directions of GLCM (i.e. horizontal and vertical directions, left and right diagonal directions). The total 13 texture features calculated from the GLCM in this study included angular second moment, the contrast, correlation, the sum of square, the inverse difference moment, the sum average, the sum variance, the sum entropy, entropy, the differential variance, the difference entropy, the information measurement of correlation 1 and the information measurement of correlation 2. A total of 26 haralick features were gained from the original protein channel (including 13 features from the averaged horizontal and vertical directions, and other 13 from the averaged left and right diagonal directions). Next, 810 ($27\times3\times10$) features were extracted after decomposing the protein segmented image into 10 levels by discrete wavelet transform (DWT) using Daubechies 1 filter. “27” indicate the 26-dimensional haralick features and 1-dimensional energy feature obtained on each of the three detailed coefficients sets at each decomposition level. Finally, after integrating the previous 26 harelick features, we obtained 836-dimensional haralick texture features per image, referred to as Har.

## **Local binary pattern (LBP)**

The local features in the patches of protein image are difficult to be reflected by global descriptors. Local descriptors, therefore, can be used as a complement to the global features. LBP (Ojala, et al., 1996; Ojala, et al., 2002) is one such local descriptor that is simple yet efficient (simple computation, insensitive to light intensity). Besides, the LBP operator can be easily used in combination with other image descriptors.

LBP calculates the gray values of the center pixel with the gray values of the neighboring pixels and a given threshold. The mathematical description of LBP is:

$$LBP_{M,R}=\sum_{m=0}^{M-1} s\left(d\right)2^{m}$$

where $d=q_{m}-q_{c}$in the function $s(d)$is the difference between the gray levels of the center pixel $q_{c}$ and the neighborhood pixel $q_{m}$. $M$is the neighboring pixels and $R$is the radius of the circular region in the neighborhood. The function $s(d)$is expressed as:

$$s\left(d\right)=\left\{_{0,\text{ }otherwise}^{1,\text{ }d\text{ }\geq\text{ 0}}\right.$$

where $d$ is set to 1 if the intensity value of the neighboring pixel is larger than or equal to the threshold, and 0 otherwise.

The LBP features are described by a histogram of binary patterns calculated over the neighborhood. In this work, 256 histograms of regions were calculated and accordingly 256-dimensional LBP features were obtained based on $M=8$ and $R=1$.

## **Completed local binary pattern**

Completed Local Binary Pattern (CLBP) proposed in (Guo, et al., 2010) calculates three components to represent the local region: the center pixel, the different sign, and the difference magnitude. The operator CLBP_Center or CLBP_C is defined for the center pixel that encodes the center pixel and converts to the binary code by global thresholding. Mathematically, CLBP_C can be defined as:

$$CLBP\_C_{M,R}=t\left(q_{c},c_{l}\right)\text{ }t\left(x,c\right)=\left\{_{0,\text{ }x<c}^{1,\text{ }x\geq c}\right.$$

where is the value of the center pixel and threshold $c_{l}$ is the averaged gray level of the entire input image. $M$is the number of the involved neighbors and $R$is the neighborhood radius.

The CLBP-Sign (CLBP_S) operator is defined for the different sign and is the same as the LBP. The difference magnitude component of CLBP is defined by the operator CLBP-Magnitude, abbreviated as CLBP_M. CLBP_M is expressed as:

$$CLBP\_M_{M,R}=\sum_{m=0}^{M-1} t\left(n_{m},c\right)2^{m}$$

where $n_{m}=\left|q_{m}-q_{c}\right|$. The threshold $c$is set to the mean value of $n_{m}$from the entire image. $M$, $R$, $q_{m}$, $q_{c}$and $t\left(x,c\right)$ are defined in equations (4) and (6).

The two operators CLBP_S and CLBP_M are produced from the Local Difference Sign-Magnitude Transform (LDSMT), which is calculated based on the referenced pixel and all the pixels that belong to the specified neighborhood.

All the three CLBP operators are in binary-encoded format and thus they can be combined together to form a CLBP histogram. We concatenated all the three operators and obtained the final 906 CLBP features based on the two configurations $\left(R=1,M=8\right)$ and $\left(R=2,M=16\right)$.

## **Rotation invariant co-occurrence of adjacent LBP**

The LBP descriptor does not keep the spatial relationships among binary patterns. Co-occurrence Among LBP (CoALBP) (Nosaka, et al., 2012) solves this problem by using four autocorrelation matrices. Rotation Invariant Co-occurrence of Adjacent LBP (RICLBP) (Nosaka, et al., 2013) is the modified version of CoALBP, which ensures the rotation invariance by attaching a label of rotation invariant to each CLBP pair.

RICLBP uses two parameters: the scale of LBP radius and the displacement among different LBP pairs. In our experiment, three different parameter sets (1,2), (2,4), and (4,8) were used to extract three different feature vectors from the target protein image. Each feature vector contained features with the dimension of$N_{P}\left(N_{P}+1\right)/2$. $N_{P}$ is the number of possible LBPs, $N_{P}=2^{M}$, where $M$ is the neighboring pixels in LBP. In our study, $M$ is set to 4 ($N_{P}=2^{4}=16$), and therefore, a 136-dimensional feature vector would be obtained for each parameter set. Finally, we integrated all three extracted feature vectors into our final proposed feature vector of 408 (136 × 3) dimension.

## **Locally encoded transform feature histogram**

Locally Encoded Transform Feature Histogram (LETRIST) proposed by Song *et al*. (Song, et al., 2018) is a simple, low dimensional yet efficient descriptor to represent an image. The LETRSIT descriptors (referred to as LET in this study) encode the mutual information within a target image across features and scale space.

First, transform features set that describes the local texture structures and the correlation among them were constructed and then quantized into texture codes. Next, the cross scale joint coding was applied to these texture codes to construct three histograms. Finally, these histograms were concatenated to generate the final 413-dimensional feature vector. The experimental setup used here was the same as (Song, et al., 2018).

# **Text S3: Stepwise discriminant analysis**

In order to select the subsets of features from the original base features, SDA was applied individually on each feature set including SLFs, LBP, CLBP, LET and RICLBP. SDA works under the assumption to distinguish various groups (classes) from one another while keeping the individual group at the same time as tightly wrapped as possible. The criterion used to measure this property is Wilks’ $\Lambda$ which can be formally defined as

$$\Lambda(p)=\frac{\left|W(X)\right|}{\left|T(X)\right|},\text{ }X\text{=}\left[x_{1},x_{2},...,x_{p-1},x_{p}\right]$$

where $X$ represents a vector with $p$ features currently used; $W$ and $T$are the within-classes covariance and the total covariance matrices, respectively, defined as

where $i$ represents the feature at *i*-th position;$j$represents the *j*-th features; $G$ is the total classes and $r$is one of the $G$class; $N_{r}$ represents all the data samples in the class $r$; $x_{irk}$ is the *i*-th and $x_{jrk}$ is the *j*-th feature values, respectively, of the data sample $k$in the class $r$; $\bar{x}_{jr}$ and $\bar{x}_{ir}$ are the mean values of *j*-th and *i*-th features in the *r*-th class; $\bar{x}_{i}$ represents the mean value of *i*-th feature over all the classes and the same as $\bar{x}_{j}$.

In order to further enhance the discriminative capability, an additional feature $x_{p+1}$ is included in the feature vector $X$in equation 8. This results the partial Wilks’ ($\Lambda\left(p+1\right)$) which can be defined as:

$$\Lambda(p+1)=\frac{\Lambda\left(\left[x_{1},x_{2},...,x_{p},x_{p+1}\right]\right)}{\Lambda\left(p\right)},\text{ }x_{p+1}\text{ is the }\left(p+1\right)\text{-th feature}$$

In order to determine whether the new feature $x_{p+1}$ will increase the discrimination significantly, the F-statistic approach is used to evaluate the significance level of the feature. The larger values of F-statistic indicate that the features have better discriminative capability. This F-statistic is known as F-to-enter or $F_{enter}$ criterion because it indicates whether $x_{p+1}$ should be included in the current feature set. On the other hand, F-to-remove or $F_{remove}$ criterion indicates whether an already included feature in current feature set should be taken out. These criteria can be defined as:

where $G$ represents all the classes and $n$ denotes the number of all data samples over all the classes; *p* is the features dimensionality currently analyzed.

In this study, the following steps were used to calculate the feature subsets from the base feature sets:

1. The within-class covariance matrix $W$ and total covariance matrix $T$ was calculated for all features.
2. F-to-remove ($F_{remove}$ ) statistic was calculated for each feature that is already included in the feature set and 1) the feature with the smallest $F_{remove}$ value and 2) level of significance (p-value) larger than a predetermined threshold was removed from the set of current features. This step was skipped when no features was yet entered.
3. $W$ and $T$ matrices were updated soon after a feature was taken out in step 2 to reflect the change.
4. F-to-enter ($F_{enter}$) statistic was calculated for each feature that was not included in the feature set and 1) the feature that had the highest $F_{enter}$ value and 2) significance level below a predetermined threshold was entered to the feature set.
5. $W$ and $T$ matrices were updated again to reflect the step 4 changes.
6. The process was stopped when no features to be added or taken out, otherwise return $W$ and $T$ to step 2.

In our study, we set the value of threshold the same as existing studies which is 0.15 (Ullah, et al., 2021; Yang, et al., 2014).

# **Text S4: Autoencoder**

An autoencoder (AE) is an artificial neural network (ANN) that can learn both the linear and non-linear relationships present in the input data space and subsequently transform them to a new data space without losing crucial information (Pierre, 2012; Rumelhart, et al., 1986). A conventional autoencoder typically consists of two main parts: 1) an encoder $h=f_{enc}(x)$, where the input data $x$ is mapped by the encoder function to a new encoded data $h$ and 2) a decoder $\hat{x}=f_{dec}(h)$, where the decoder function produces a reconstruction of the input data $x$ by mapping the encoded data $h$ to $\hat{x}$. **Figure S1** illustrates an example of a typical autoencoder, where the *n*-dimensional input data $x$ from the Input Layer is encoded in the Hidden Layer to a *q*-dimensional encoded data $h$ (i.e. Feature 1) and the encoded data $h$ is further decoded to the *n*-dimensional $\hat{x}$ in the Output Layer.


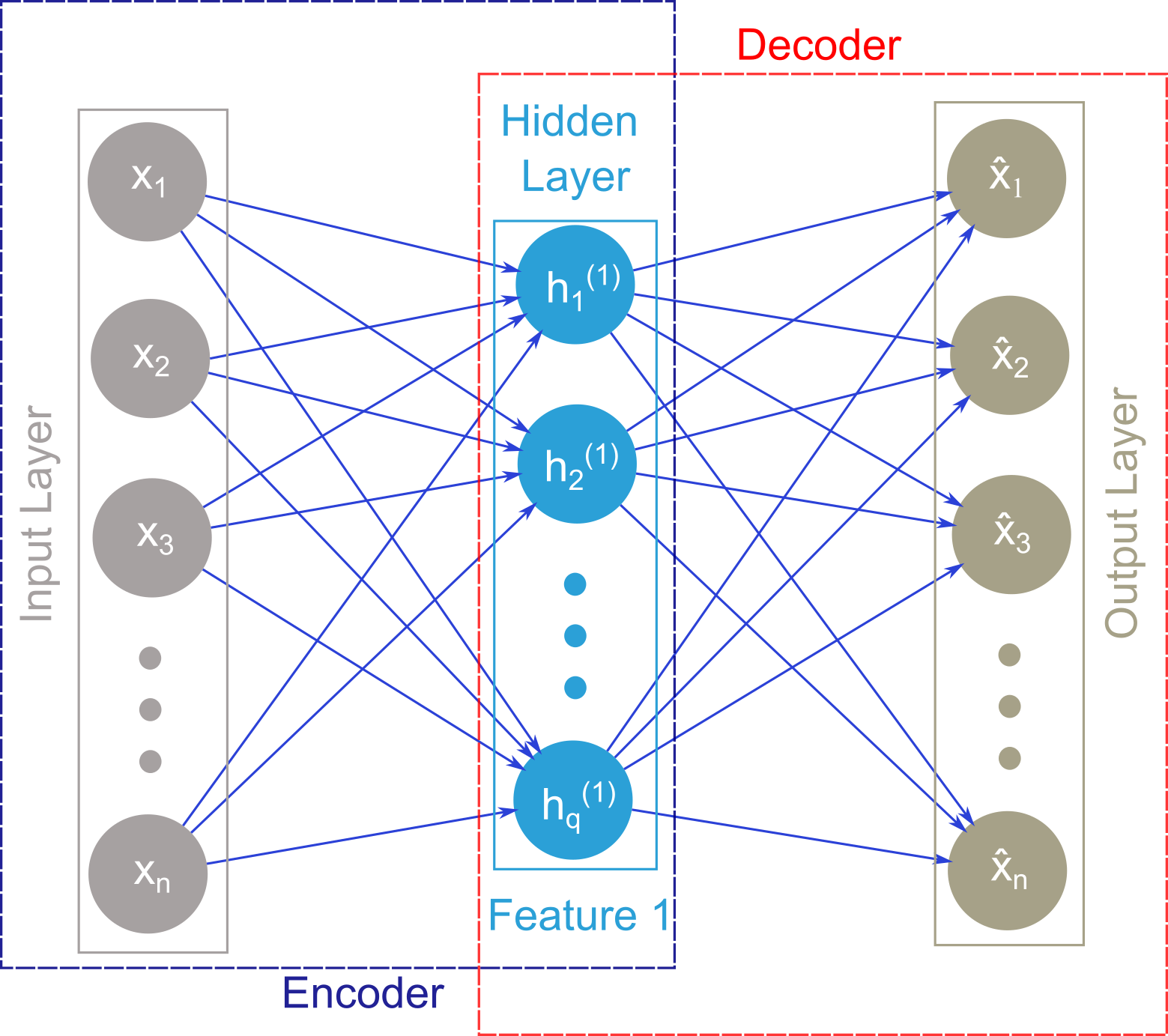


**Fig. S1.** The architecture of the conventional autoencoder.

# **Text S5: The layers size and hyperparameters of SAE-SM in 2L-SAE-SM**

The size of input layer for each of the SAE-SMs in the 1st-level is equal to the size of its particular input optimal training feature set which is fed to the first hidden layer. The first encoder produces first hidden layer activation output features with the size of 100 which is fed to the second encoder of SAE-SM. The second encoder generates the second hidden layer activation output features with the size of 50 which is further fed as an input to the SoftMax classifier. The output size of the SoftMax layer is equal to the number of all classes (subcellular locations) which are 7.

In the 2nd-level of SAE-SM, the input of size 7 is generated by mean ensemble method and is served as the input to the first encoder. Similarly, the layer 1 and 2 hidden size of the first and second encoder are 100 and 50, respectively. The output of the SoftMax classifier is the probabilities of 7 different classes.

The hyperparameter setting is kept consistent for both 1st- and 2nd-levels of the 2L-SAE-SMs. The weight decay parameter which is also called lambda is set to 2e-5. The weight of the sparsity penalty term which is denoted by beta is set to 3. The sparsity parameter which is denoted by ρ is set to 0.1.

# **Text S6: Training 2L-SAE-SM**

1. Training the 1st-level SAE-SMs

In the 1st-level of 2L-SAE-SM, we trained each of the *T* SAE-SMs using its corresponding optimal heterogeneous training feature set. We used the standard training method for training each SAE-SM. Taking the *t*-th SAE-SM as an example, the optimal heterogeneous training feature set $S_{t}^{opt}$ is provided to the SAE-SM to train the *t*-th SAE-SM, denoted as SAE-SM*_t_*.

1. Training the 2nd-level SAE-SM

In order to train the 2nd-level SAE-SM, we need to have an ‘intermediate feature’ set generated by the 1st-level. In particular, the following strategy can be used to effectively generate the ‘intermediate feature’ set:

For each image sample *j* in the training dataset $X=\{(x_{j};y_{j})\}_{j=1}^{N}$, where $x_{j}$ and $y_{j}$ are the *j*-th image sample and its corresponding label, respectively, $N$ is the total number of image samples in $X$, the *t*-th ‘intermediate decision’ vector $\tilde{p}_{j,t}$ with the dimension of *C* can be obtained by feeding the *t­*-th $(1\leq t\leq T)$ optimal heterogeneous feature vector $x_{j,t}^{opt}$ of the image sample *j* into a new trained $\text{SAE-SM}_{t}^{\text{†}}$ rather than into SAE-SM*_t_*. It is important to mention that the training feature set of $\text{SAE-SM}_{t}^{\text{†}}$ should not include the data pair $(x_{j,t}^{opt};y_{j})$. For $T$ optimal heterogeneous feature vectors of the image sample *j*, $T$ ‘intermediate decision’ vectors, denoted as $\{\tilde{p}_{j,t}\}_{t=1}^{t=T}$, can be generated. Finally, by integrating the $T$ ‘intermediate decision’ vectors by ME, the *j*-th ‘intermediate feature’ vector of ­­*C*-dimension, denoted as $\tilde{f}_{j}$, can be obtained. Let $(\tilde{f}_{j},y_{j})$ be the data pair produced from the *j*-th labeled image sample $(x_{j};y_{j})$, then for the $N$ image samples, the corresponding ‘intermediate feature’ set, denoted as $F_{ME}=\{(\tilde{f}_{j},y_{j})\}_{j=1}^{N}$, can be constructed as the training set for 2nd-level SAE-SM.

To avoid the overfitting problem, the following two different strategies can be used to train $\text{SAE-SM}_{t}^{\text{†}}$.

Strategy 1 works under the following assumption:

For each image sample *j* in the training dataset $X$, in order to generate “intermediate decision” vector $\tilde{p}_{j,t}$ for the *t*-th heterogeneous feature vector of image sample *j* (i.e. $x_{j,t}^{opt}$), an SAE-SM, denoted as $\text{SAE-SM}_{t}^{\text{†}}$, is trained on the training set $\tilde{S}_{t}^{opt}=S_{t}^{opt}-\{(x_{j,t}^{opt};y_{j})\}$ rather than training on $S_{t}^{opt}$. This process can avoid overfitting problem by ensuring that the data pair $(x_{j,t}^{opt};y_{j})$ was not used when training the corresponding $\text{SAE-SM}_{t}^{\text{†}}$.

In strategy 2, first, each of the $T$ heterogeneous feature sets is randomly divided into $K$ non-overlapping equally sized subsets and then an SAE-SM is trained on each subset. Taking the *t*-th heterogeneous feature set (i.e. $S_{t}^{opt}$) as an example, $S_{t}^{opt}$ is first partitioned into $\tilde{S}_{t,1}^{opt},\text{ }\tilde{S}_{t,2}^{opt},\text{ }...,\text{ }\tilde{S}_{t,k}^{opt},\text{ ..., }\tilde{S}_{t,K}^{opt}$ subsets. Then, on each feature set, denoted as $S_{t}^{opt}-S_{t,k}^{opt}$, an SAE-SM, denoted as $\text{SAE-SM}_{t,k}$ is trained. The symbol “$-$” denotes the set difference in the set theory. Once the training is done, then for each image sample *j* in the training dataset $X$, we can generate “intermediate decision” vector $\tilde{p}_{j,t}$ by feeding the *t*-th heterogeneous feature vector of image sample *j* (i.e. $x_{j,t}^{opt}$) into an $\text{SAE-SM}_{t,k}$, denoted as $\text{SAE-SM}_{t}^{\text{†}}$, trained on feature set $S_{t}^{opt}-S_{t,k}^{opt}$ such that $(x_{j,t}^{opt};y_{j})\in S_{t,k}^{opt}$, and selected from the pre-trained SAE-SM set $\{\text{SAE-SM}_{t,k}\}_{k=1}^{k=K}$.

In order to generate the “intermediate feature” set by applying the strategy 1, which is simply known as the leave-one-out cross validation (LOOCV), $T\times N$ SAE-SMs need to be trained, where *T* is the total number of heterogeneous features extracted from each image sample and *N* is the total number of image samples in the training dataset *X*. Considering that our benchmark training dataset had 2708 image samples and we extracted five different features from each image sample, the total number of SAE-SMs that need to be trained was $13540=5\times2708$. By applying the strategy 2 which is also known as the *k*-fold cross-validation, $T\times K$ SAE-SMs can be trained, where $K$ is the total number of the subsets. Here, in our study, we set $K$ to be 10, then by adopting strategy 2, a total of $50=5\times10$ SAE-SMs were trained on our benchmark training dataset. Strategy 1, which trained 13540 SAE-SMs in our case, is time-consuming and might fail to work. Therefore, strategy 2, which substantially reduces the number of training SAE-SMs to 50 and avoids the overfitting problem as well, was adopted in this study.

# **Text S7: Pipeline of the proposed method**

In this study, we developed a new bioimage-based pipeline termed PScL-2LSAESM for protein subcellular localization prediction, in which a two-level SAE-SM called 2L-SAE-SM is used to efficiently integrate multiple heterogeneous feature sets.

In the training phase, for each IHC input image, PScL-2LSAESM first separates the image into DNA and protein channels by calling the corresponding image separation program (Image Separation) and then extracts its multiple heterogeneous features including SLFs-Raw, LBP-Raw, CLBP-Raw, LET-Raw and RICLBP-Raw (Feature Extraction). Next, by calling the SDA algorithm, more discriminative features (i.e. the optimal heterogeneous features) are selected from each heterogeneous feature set (Feature Selection). Then, the selected five optimal heterogeneous feature sets (i.e. SLFs-Optimal, LBP-Optimal, CLBP-Optimal, LET-Optimal and RICLBP-Optimal) are sent to train their corresponding 1st-level SAE-SM model. To generate $F_{ME}$, the outputs of the newly trained SAE-SMs via 10-fold cross-validation are ensembled using the ME method and further sent to 2nd-level SAE-SM to train the proposed engine as the prediction model (2L-SAE-SM). At the prediction stage, for a given protein image from the testing data, the strategies utilized in the training phase are used to generate five optimal heterogeneous features; afterward, these five optimal heterogeneous features are then fed to the corresponding trained 1st-level SAE-SMs. Next, the 1st-level SAE-SMs outputs are ensembled using the ME method and then sent to trained 2nd-level SAE-SM to generate the final prediction result (i.e. Prediction).

PScL-2LSAESM is a single-label multiclass model for protein subcellular localization prediction which is trained on a newly collected benchmark training dataset PScL2708 from the HPA data bank, encompassing the samples for seven major subcellular locations.

# **Text S8. Software and hardware configuration settings for model construction and experiments**

The experiments of the proposed method and other comparative classifiers in the paper were performed by using the advanced version of R2019a of Matrix Laboratory (MATLAB) software and Spyder which is and integrated development environment (IDE) for python. We implemented all the algorithms i.e. feature extraction methods, features selection method, feature integration and classifier of the proposed system by employing written MATLAB scripts. The other classifiers were implemented in python and the performance evaluation in terms of all the metrics were also calculated using the python scripts and its libraries. The proposed model was also tested on the MATLAB version R2017a.

The hardware requirements for the implementation and experiments are:

Processor: Intel ® Core™ i7-4720HQ

CPU: 2.60 GHz (8 CPUs), ~2.6 GHz

Installed Memory (RAM): 16.0GB

GPU: NVIDIA GeForce GTX 960M with 4GB of dedicated memory

Windows: 10 (64-bit Operating System)

# **Text S9. Performance evaluation matrices**

The Rec_M_, Prec_M_ and F1-Score_M_ are described as:

where $j$ represents the *j*-th class in the total number of $G$classes. $recall_{(j)}$, $precision_{(j)}$ and $F1_{(j)}$ are the recall, precision and F1-score of *j*-th class, respectively, computed as:

where $TP_{(j)}$, $FN_{(j)}$ represents true positive and false negative predictions in class *j*, respectively; $FP_{(j)}$ (false positive) represents the samples in the class of *j* that are wrongly predicted into class *j*. Suppose the prediction results for the $G$ classes can be represented as a $G\times G$confusion matrix $C$, where $C_{gg}$ is the number of truly predicted samples in class $k$. Let $C_{gj}$ denotes the number of data samples predicted to be in the class $g$ while in reality belongs to class $j$, $g\neq j$. For the *j*-th class, its corresponding true positive ($TP_{(j)}$), false positive ($FP_{(j)}$) and false negative ($FN_{(j)}$) can be calculated according to confusion matrix.

Similarly, the OA can be calculated as:

$$\text{OA}=\frac{\sum_{j}^{G} TP_{(j)}}{P}$$

where $\sum_{j}^{G} TP_{(j)}$ represents the total number of data samples correctly predicted;$P$ denotes the total number of data samples being predicted. The MCC can be calculated as:

$$\text{MCC}=\frac{\sum_{g,j,r=1}^{G} C_{gg}C_{jr}-C_{gj}C_{rg}}{\sqrt{\sum_{g=1}^{G} \left(\sum_{j=1}^{G} C_{gj}\right)\left(\sum_{g',j'=1|g'\neq g}^{G} C_{g'j'}\right)}\sqrt{\sum_{g=1}^{G} \left(\sum_{j=1}^{G} C_{jg}\right)\left(\sum_{g',j'=1|g'\neq g}^{G} C_{j'g'}\right)}}$$

The MCC for multiclass is computed by taking all the entries of $C_{G\times G}$ involving all the $G$ classes into account.

# **Text S10. Performance comparison of different classifiers based on serial integration of the optimal heterogeneous feature sets**

To evaluate and compare the discriminative capabilities of SAE-SM and other classifiers, we first generated a new feature set by serially integrating all the five optimal heterogeneous feature sets obtained via the SDA feature selection algorithm and then fed it to the RF, eXtreem Gradient Boosting (XGBoost) (Chen and Guestrin, 2016), Light Gradient Boosting Machine (LGBM) (Ke, et al., 2017), SVM and SAE-SM. The performance comparison between all these classifiers is provided in **Table S1**. The experimental results in **Table S1** were generated by performing 10-fold cross-validation on the entire PScL2708 dataset.

**Table S1.** Performance comparison of different classifiers on 10-fold cross-validation using the benchmark training dataset PScL2708.

| Method | OA (%) | Rec_M_ (%) | Prec_M_ (%) | F1-Score_M_ | MCC |
| --- | --- | --- | --- | --- | --- |
| Serial+RF | 71.30 | 68.05 | 73.46 | 0.6931 | 0.6617 |
| Serial+XGBoost | 80.90 | 79.23 | 80.49 | 0.7974 | 0.7747 |
| Serial+LGBM | 81.94 | 80.11 | 81.73 | 0.8074 | 0.7869 |
| Serial+SVM | 84.26 | 83.35 | 83.91 | 0.8358 | 0.8145 |
| Serial+SAE-SM | 87.18 | 86.52 | 86.56 | 0.8653 | 0.8489 |

From **Table S1**, we can see that the SAE-SM performed well than the other classifiers on the serially integrated feature set. SAE-SM achieved the OA, F1-Score_M_ and MCC of 87.18, 0.8653 and 0.8489, respectively, which were about 2.92-15.88%, 2.95-17.22% and 3.44-18.72% higher than those of the other classifiers listed in **Table S1**. More specifically, the OA, F1-Score_M_ and MCC of SAE-SM were increased by 2.92%, 2.95% and 3.44%, respectively, compared with the runner-up SVM classifier. In terms of the other evaluation indices (i.e. Rec_M_ and Prec_M_), SAE-SM also outperformed the runner-up SVM model and all the other models.

We further evaluated and compared the predictive performance of these classification models in terms of meanAUC, meanAUPR, stdAUC and stdAUPR. The results are shown in **Figure S2(A)-(D)**. Accordingly, we conclude that the SAE-SM consistently achieved better performance than the runner-up SVM and other classification models in terms of meanAUC, meanAUPR, stdAUC and stdAUPR. In addition, from **Figure S2(C)**, we can also see that XGBoost and LGBM classifiers performed better than the runner-up SVM in term of stdAUC; however, amongst all these compared classifiers, SAE-SM clearly outperformed the others with a larger margin.


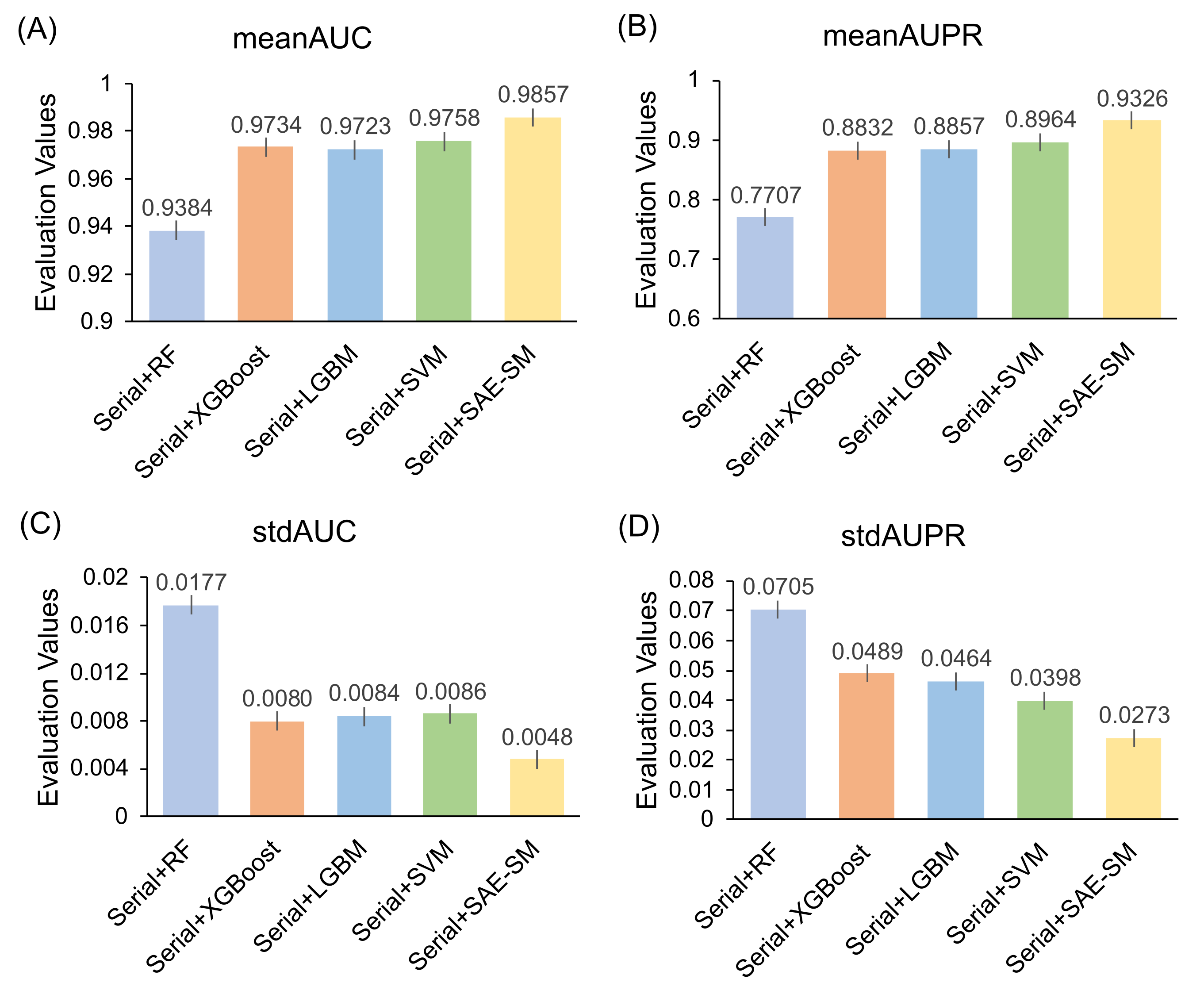


**Fig. S2.** Performance comparison of different classification models. Panels (A), (B), (C) and (D) illustrate the performance comparison in terms of meanAUC, meanAUPR, stdAUC and stdAUPR, respectively.

Summarizing the performance results provided in **Table S1** and **Figure S2(A)**-**(D)**, we conclude that the proposed SAE-SM classification model outperformed the other classification models and thus provides a better choice for image-based protein subcellular localization prediction in human tissues.

# **References**

Chen, T. and Guestrin, C. Xgboost: A scalable tree boosting system. In, *Proceedings of the 22nd ACM SIGKDD international conference on knowledge discovery and data mining*. San Francisco, California, USA: Association for Computing Machinery, New York, NY, USA; 2016. p. 785-794.

Guo, Z., Zhang, L. and Zhang, D. A completed modeling of local binary pattern operator for texture classification. *IEEE Transactions on Image Processing* 2010;19(6):1657-1663.

Ke, G.*, et al.* LightGBM: a highly efficient gradient boosting decision tree. In, *Proceedings of the 31st International Conference on Neural Information Processing Systems*. Long Beach, California, USA: Curran Associates Inc.; 2017. p. 3149–3157.

Newberg, J. and Murphy, R.F. A Framework for the automated analysis of subcellular patterns in human protein atlas images. *Journal of Proteome Research* 2008;7(6):2300-2308.

Nosaka, R., Ohkawa, Y. and Fukui, K. Feature extraction based on co-occurrence of adjacent local binary patterns. In: Ho, Y.-S., editor, *Advances in Image and Video Technology*. Berlin, Heidelberg: Springer Berlin Heidelberg; 2012. p. 82-91.

Nosaka, R., Suryanto, C.H. and Fukui, K. Rotation invariant co-occurrence among adjacent LBPs. In: Park, J.-I. and Kim, J., editors, *Computer Vision - ACCV 2012 Workshops*. Berlin, Heidelberg: Springer Berlin Heidelberg; 2013. p. 15-25.

Ojala, T., Pietikäinen, M. and Harwood, D. A comparative study of texture measures with classification based on featured distributions. *Pattern Recognition* 1996;29(1):51-59.

Ojala, T., Pietikainen, M. and Maenpaa, T. Multiresolution gray-scale and rotation invariant texture classification with local binary patterns. *IEEE Transactions on Pattern Analysis and Machine Intelligence* 2002;24(7):971-987.

Pierre, B. Autoencoders, Unsupervised Learning, and Deep Architectures. In.: PMLR; 2012. p. 37-49.

Rumelhart, D.E., Hinton, G.E. and Williams, R.J. Learning Internal Representations by Error Propagation. In: Rumelhart, D.E. and McClelland, J.L., editors, *Parallel Distributed Processing: Explorations in the Microstructure of Cognition, Volume 1: Foundations*. Cambridge, MA: MIT Press; 1986. p. 318--362.

Song, T.*, et al.* LETRIST: Locally encoded transform feature histogram for rotation-invariant texture classification. *IEEE Transactions on Circuits and Systems for Video Technology* 2018;28(7):1565-1579.

Ullah, M.*, et al.* PScL-HDeep: image-based prediction of protein subcellular location in human tissue using ensemble learning of handcrafted and deep learned features with two-layer feature selection. *Briefings in Bioinformatics* 2021;22(6).

Yang, F.*, et al.* Image-based classification of protein subcellular location patterns in human reproductive tissue by ensemble learning global and local features. *Neurocomputing* 2014;131:113-123.
